# LeGO-3D: 3D imaging of lung metastases and vascularisation using light sheet fluorescence microscopy

**DOI:** 10.1038/s44303-025-00111-0

**Published:** 2025-11-07

**Authors:** Sabrina M. Lewis, Jean Berthelet, Lachlan W. Whitehead, Pradeep Rajasekhar, Farrah El-Saafin, Caroline Bell, Shalin Naik, Delphine Merino, Verena C. Wimmer, Kelly L. Rogers

**Affiliations:** 1 Walter and Eliza Hall Institute of Medical Research, Parkville, VIC Australia; 2 Department of Medical Biology, University of Melbourne, Parkville, VIC Australia; 3 Olivia Newton-John Cancer Research Institute, Heidelberg, VIC Australia; 4 School of Cancer Medicine, La Trobe University, Bundoora, VIC Australia; 5 Austin Health, Heidelberg, VIC Australia; 6 Present Address: Heidelberg University Hospital, Heidelberg, Germany

**Keywords:** Microscopy, Light-sheet microscopy, Biological techniques, 3-D reconstruction, Fluorescence imaging, Optical imaging, Cancer, Cancer imaging, Image processing

## Abstract

Cancer metastasis involves a complex cascade of events, where cancer cells migrate from their site of origin to secondary sites via the lymphatic and circulatory system. During this process, some cancer subclones will successfully ‘seed’ at distant organs to generate lethal metastases. Here, we optimised a method for tracking cancer cells in metastatic breast cancer tumours and investigated their complex interplay with the lung vasculature using lentiviral-based optical barcoding (LeGO). Given the regional heterogeneity in lung tissue microenvironments as well as lobar asymmetry, we used light sheet microscopy to perform three-dimensional (3D) imaging of wholemount lung lobes. The results revealed that polychromatic metastases occurred less frequently than monochromatic metastases and were more likely to be located nearer to blood vessels in both spontaneous (i.e. mammary fat pad injections) and experimental (i.e. tail vein injections) mouse assays of metastasis. This 3D imaging and analytic pipeline can provide unique insights about metastatic heterogeneity and dynamics, and represents a new avenue for studying therapeutic response across large volumes of lung tissue.

## Introduction

Metastatic disease is associated with poor clinical outcomes in breast cancer patients^1^. This can be attributed to the complexity of the disease, which involves a cascade of cellular migratory events. During metastasis, breast cancer cells will invade surrounding tissue before spreading to the lymph nodes. Cancer cells will then enter the vascular or lymphatic systems (a process known as intravasation)^2^, circulate in blood vessels, and then invade and seed at secondary sites (a process known as extravasation)^3^.

The tumours that give rise to metastases are composed of cellular subpopulations with highly variable genomic and non-genomic features^4^. Subpopulations of cells originating from a common ancestral cell within a tumour are typically referred to as ‘clones’. Previous studies have shown that individual clones may have distinct properties, including differences in respect to their ability to proliferate and disseminate to different organs^5,6^, and resist therapy^7^.

Individual lung metastases have been shown to be composed of a single clonal population (monoclonal) or multiple clonal populations (polyclonal)^8–12^. Here, clonal cooperation or competition may be involved, which has implications for cancer cell survival^13^. Understanding the molecular properties of these cells and their behavior, such as why some clones grow more aggressively in certain microenvironments, or how these clones interact with each other and the blood vessels, can provide critical insights for the prevention or treatment of metastasis.

Several approaches have been developed to study cancer diversity and clonal interactions^14^. Among these, preclinical models using mouse or human cancer cell lines are commonly used to gain insight into metastatic processes. Notably, optical or genetic barcoding approaches have been developed to spatiotemporally track individual cancer clones in vivo^15–17^. One such optical barcoding method, known as Lentiviral Gene Ontology vectors (LeGO), is based on lentiviral transduction of host cell lines with up to six different fluorescent reporters^18,19^. An advantage of this approach is that it leads to the combinatorial expression of fluorescent proteins in individual cells, allowing lineage tracing of multiple clonal populations using various microscopy techniques^18,20,21^. We previously described that the combination of 5 fluorescent tags: BSVTK (eBFP2, tSapphire, Venus, tdTomato and Katushka) leads to the robust detection of 31 individual cancer clones in multiple organs, by confocal imaging and flow cytometry^11^. However, this and other similar approaches have only enabled smaller tissue volumes to be imaged with a reasonable degree of throughput, using confocal or multiphoton microscopy^22,23^. To our knowledge, tissue clearing and light sheet microscopy have not been used in combination to generate whole mount tissue images containing LeGO-labelled cells.

Here, we used a 3 colour LeGO system to visualise 7 barcoded populations and combined this with tissue clearing and light sheet microscopy to enable three-dimensional (3D) imaging of cancer cells in wholemount tissues, such as the lung and brain. We optimised this method together with vessel casting^24^ and applied a semi-automated image analysis pipeline to track and quantify the anatomical relationship of metastatic populations to the lung vasculature in large 3D datasets. This approach enabled the spatial organisation and behaviour of metastatic populations to be studied in whole lung lobes, which is a common site of metastasis in patients with late-stage breast cancer^25^. We also compared the number and anatomical distribution of mono- and polychromatic metastases in both intravenous (experimental metastasis assay) and mammary fat pad (spontaneous metastasis assay) injected mouse assays. We found that polychromatic metastases were closer in proximity to blood vessels than their monochromatic counterparts, regardless of the mode of injection. This 3D multidimensional imaging approach therefore expands the repertoire of experimental models for understanding cancer cell dynamics in large-scale tissue microenvironments.

## Results

### A method for anatomical tracking of metastatic cancer cell populations in wholemount lung lobes

**Fig. 1:**
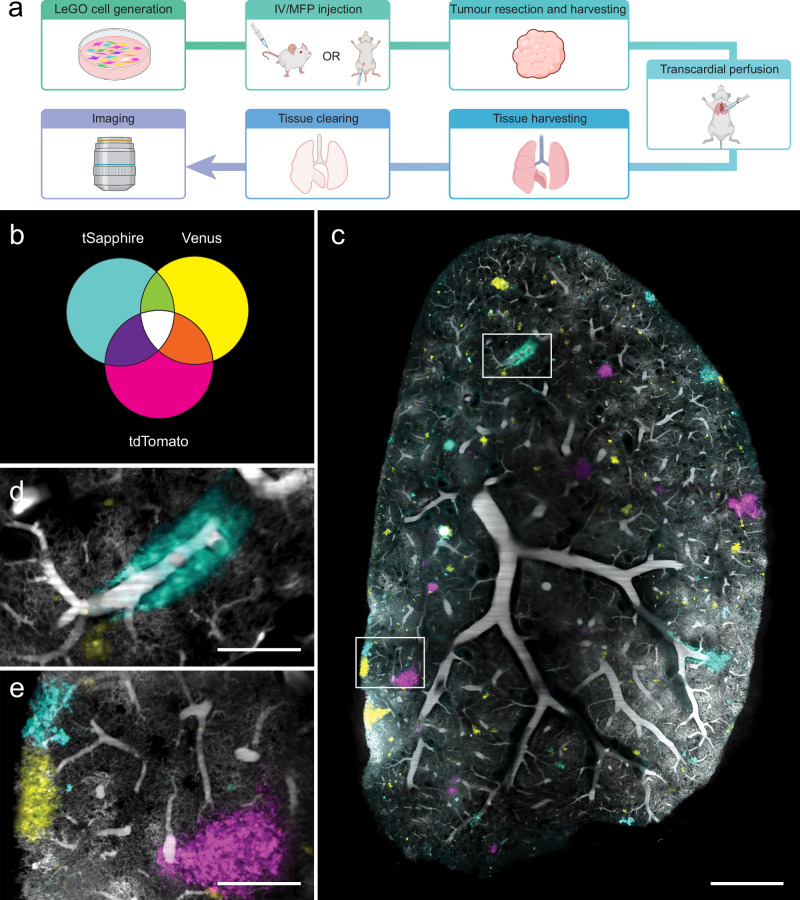
Tissue clearing, blood vessel and metastasis visualisation in murine lungs. **a** Streamlined workflow for SVT BP tracking and vessel casting in large tissue volumes. Created in BioRender. Lewis, S. (2025) https://BioRender.com/2abbxqj. **b** Venn diagram representing seven unique combinations for cancer cell populations tracking by expression of three fluorescent proteins: tSapphire, Venus and tdTomato. **c** Representative light sheet image of vessel cast vasculature (grey) and SVT metastases in lungs. Scale bar 1000 μm. **d**, **e** Magnified regions from white boxes in (**c**) are shown. Scale bar 250 μm.

To investigate the distribution of breast cancer cell clonal populations relative to an anatomical reference such as the vasculature, we optimised tissue clearing together with a method for labelling blood vessels and a strategy to optically barcode cancer cells for light sheet microscopy (Fig. 1a).

We generated MDA-MB-231 cells stochastically expressing one, two or three fluorophores (tSapphire, Venus and tdTomato, referred to below as combinatorial SVT), which produces seven different traceable cell populations (Fig. 1b). Here, as these cancer subpopulations were not derived from single cell barcoded cells, they are referred to as ‘barcoded populations’ (BPs) rather than clones. After in vitro expansion of BPs, 100,000 cells were delivered intravenously (IV) or transplanted into a murine mammary fat pad (MFP) (Fig. 1a). Primary tumours generated via MFP transplantation were resected approximately one month after initial transplantation, before they reached 500 mm^3^. In both groups, mice were humanely euthanised when they reached ethical endpoint. To ensure optimal preservation of the tissue, transcardial perfusions were performed, followed by vessel casting^24^ to fluorescently label blood vessels and capillaries with BSA-conjugated Alexa 647 (see methods for details), and tissue clearing (Supplementary Fig. 1a–d). To ascertain how well vessel casting labelled the blood vessel network, we used transgenic Flk1-GFP mice that constitutively express GFP in endothelial cells^26^. Following perfusion and vessel casting, Flk1-GFP brains were sectioned, cleared and imaged using confocal microscopy (see methods). These experiments confirmed that GFP-expressing endothelial cell-lined vessels overlapped with BSA-Alexa 647 labelled vessels (Supplementary Fig. 1e–j), though some Flk1-GFP positive capillaries were observed to be negative for Alexa 647, and vice versa (Supplementary Fig. 1e–j). This is in agreement with a previous study, where immunohistochemistry staining overlapped with vessel casting^27^.

To confirm that vessel casting could be combined with combinatorial SVT labelled metastases (Fig. 1b), and did not interfere with downstream tissue processing, we cleared vessel-cast wholemount lung using passive clarity technique (PACT), an aqueous-based tissue clearing technique^28^. The cleared organs were then imaged in 3-dimensions (3D) using a Zeiss Z.7 light sheet microscope (Fig. 1c), which provides a lateral resolution of ~1.2 μm and axial resolution of ~3 μm. Blood vessel labelling was maintained throughout the clearing process and all seven combinatorial SVT populations could be identified (Fig. 1c–e).

### A semi-automated, machine learning based pipeline for analysing the distribution of barcoded populations and blood vasculature in whole lung lobes

**Fig. 2:**
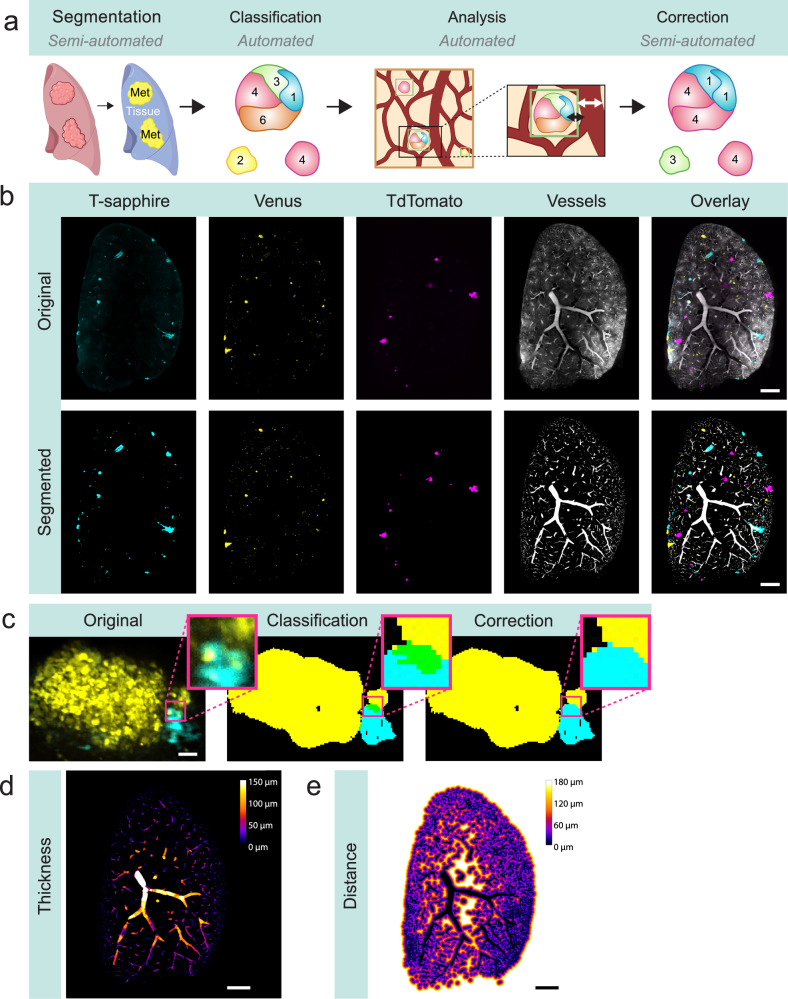
LeGO metastasis and vessel analysis pipeline. **a** Analysis workflow using semi-automatic and automatic Fiji Macro and Python scripts. Created in BioRender. Lewis, S. (2025) https://BioRender.com/g0il646. **b** Each image channel is segmented using Ilastik. Manual training is performed before automatic interpolation across the entire lung is completed. Representative image of a metastasis-containing lung, acquired on a light sheet microscope. TSaphhire (cyan), Venus (yellow), TdTomato (magenta) and blood vessel (grey) channels, as well as overlay image is shown. Original and segmented image is shown. Scale bar 1000 μm. **c** LeGO BPs are automatically classified across thousands of metastases in lungs. Representation SVT metastasis (left), LeGO BPs classification of SVT metastasis (middle) and LeGO BPs correction of SVT metastasis (right). Scale bar, 50 μm. **d**, **e** Automatic measurement of the metastasis’ nearest vessel and diameter of that vessel is performed. **d** Representative local thickness map shows diameter of vessels in lung. Brightest pixels represent larger vessel diameters, whereas dimmer pixels represent smaller vessel diameters (μm). White pixels represent vessels that are 150 μm or larger. Scale bar, 1000 μm. **e** Representative local distance map shows distance to vessels in lung. Brightest pixels represent nearer vessels, whereas dimmer pixels represent further vessels. White pixels represent distances that are 180 μm or further. Scale bar, 1000 μm.

We created a multiparametric and bespoke analysis pipeline for the measurement of combinatorial SVT population frequencies, metastasis numbers and volume, vessel diameter and distance, as well as surface areas from each of the large 3D datasets generated (~0.5 terabytes in size) (Fig. 2a, Supplementary Fig. 2). To do so, we used ilastik, an open-source machine learning software, to train a pixel classifier that detects the metastatic area in the image^29^. Segmentation masks were generated using Fiji (Fig. 2b) and tSapphire, Venus and tdTomato segmented channels were combined to represent all 7 colours of metastases in the tissue.

An automated Python script was then run for classification of combinatorial SVT BPs across all filtered metastases. Classification was performed by “bitwise encoding” of the masked channels: channel 1 was assigned the binary value 001, channel 2 as 010, and channel 3 as 100. Summing the masks produced a map of all possible binary combinations (‘barcodes’), and the proportion of each barcode was quantified and reported in the output spreadsheet. Whilst fully automated pipelines are fast and reproducible, they can be less effective at accurately deciphering non-binary information, such as the SVT BPs in metastases. Therefore, we used a semi-automated Fiji macro script to determine the accuracy of the SVT BPs identified in metastases during the initial steps of classification. The segmentation and classification of BPs, especially those derived from multiple fluorescent proteins, required further correction in some cases (Fig. 2c). This was due to variations in fluorophore brightness and occasional overlap with neighbouring BPs, which led to the misidentification of some populations. To overcome these issues, we used our semi-automated Fiji script to correct the misidentified BPs by manually assigning them to their correct colour. In ambiguous cases, colour assignment was made according to fluorescence intensity and in the context of BPs in the surrounding tissue.

Next, we sought to use this dataset to further explore the relationship between different metastatic cell populations (based on the identification of combinatorial SVT identities) in the lung, and their relationship with the lung vasculature. Here, we aimed to measure several key features of the blood vessels, such as the diameter of the nearest vessel to each metastasis (defined as any SVT labelled object i.e. a cancer cell or group of cells). A local thickness map was used to calculate vessel diameters (Fig. 2d), and a distance map approach was used to measure the edge-to-edge distances between the metastasis and its closest blood vessel (Fig. 2e). We also measured the total surface area of a metastatic tumour which intersects with that of a blood vessel using a custom automated Python script. All custom scripts can be found on Github: https://github.com/BioimageAnalysisCoreWEHI/lego_analysis/tree/main/data_analysis. Importantly, we validated this pipeline as an appropriate method to quantify a wide range of parameters, including but not limited to, object volumes, diameters, object to object distances and localisations in large three-dimensional volume datasets (>0.5 TBs), in a variety of different experimental settings.

### A quantitative comparison of lung metastases in MFP- and IV-injected metastasis assays

**Fig. 3:**
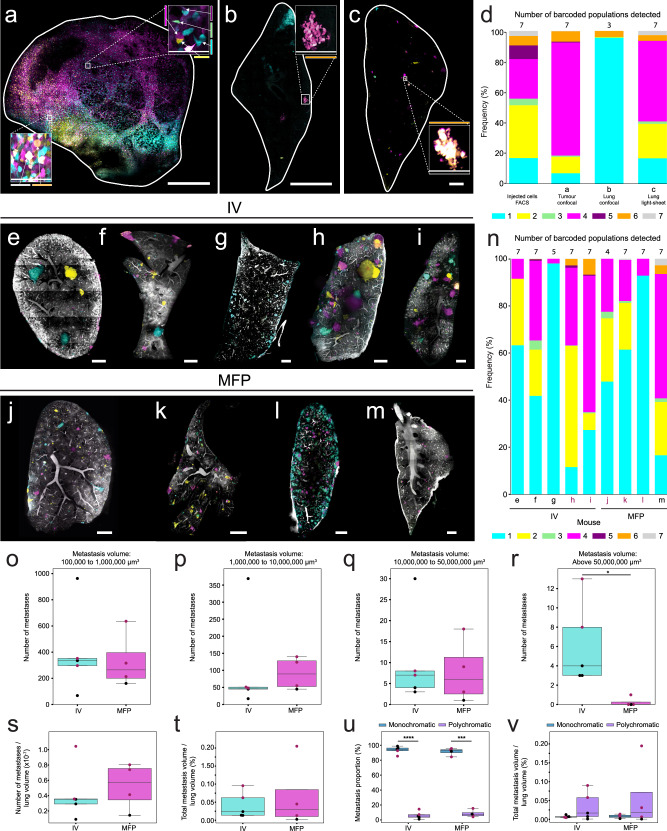
Barcoded population frequencies and metastatic burden of MDA-MB-231 cells in primary tumours and lung tissue. **a** Matched primary tumour, **b** confocal lung lobe section and **c** light sheet lung lobe. Scale bar 1000 μm. **a**–**c** Magnified regions from white boxes. Scale bar **a** 90 μm, **b**, **c** 180 μm. Arrows indicate different BPs. **d** Stacked bar chart shows biomass of seven BPs in injected cells, primary tumour and lung from one animal. BP biomasses were determined by flow cytometry (injected cells) or from imaging data (tumour, lung). Lungs from (**e**–**i**) intravenously (IV) injected and (**j**–**m**) mammary fat pad (MFP) injected mice. Scale bar 1000 μm. **n** Stacked bar chart shows contribution of seven BPs to metastasis biomass in IV and MFP lungs. BP biomasses were determined from imaging data. **d**, **n** Number of BPs is shown above each bar. Key is shown below stacked bar chart, where each colour represents a unique BP. Number of metastases with volume **o** 100,000 μm^3^ to 1,000,000 μm^3^ (100–1000 cells, small), **p** 1,000,000 μm^3^ to 10,000,000 μm^3^ (1000–10,000 cells, medium), **q** 10,000,000 μm^3^ to 50,000,000 μm^3^ (10,000–50,000 cells, large), **r** above 50,000,000 μm^3^ (>50,000 cells, very large) in IV and MFP mice. Independent *t*-test, P* = 0.030645. **s** Number of metastases normalised to lung volume in IV and MFP mice. **t** Percentage metastasis volumes to lung volumes in IV and MFP mice. **u** Proportion of monochromatic and polychromatic metastases in IV and MFP mice. Paired *t*-test, P**** = 0.000043, P*** = 0.000450. **v** Percentage metastasis volumes to lung volumes of monochromatic and polychromatic metastases in IV and MFP mice. All box plots show the median and interquartile range (IQR), with lines representing 1.5*IQR. *n* = 5 mice for IV and *n* = 4 mice for MFP from 4 independent experiments. The number of monochromatic metastases analysed for IV-injected mice ranged from 384 to 5300, and for MFP-injected mice ranged from 1057 to 3196. The number of polychromatic metastases analysed for IV-injected mice ranged from 21 to 419, and for MFP-injected mice ranged from 58 to 219. In o-r, subsets of metastases were analysed based on metastasis volume. For one experiment, mice from the IV and MFP groups were injected in parallel (highlighted by the burgundy-coloured dots).

We also compared the BPs in a primary tumour (Fig. 3a) and lung section (Fig. 3b) using confocal microscopy, to a matched lung from the same animal acquired via light sheet microscopy (Fig. 3c). Confocal images were analysed using manual thresholding and light sheet images were analysed using the pipeline described above. BP frequencies were compared to frequencies in the original injected cell line, which were analysed via flow cytometry (Fig. 3d). All colours were present in the injected population and primary tumour, with similar frequency observed in primary tumours and lung metastases, as previously described^11,30^. However, the number and frequency of BPs detected by confocal imaging of one tissue section from a lung, differed to what was measured in an entire lung lobe using light sheet microscopy. This reflects the heterogenous and non-uniform nature of lung tissue, which implies that there will be variations in the spatial distribution of cells according to where tissue is sampled (Fig. 3d).

We next compared the heterogeneity of lung metastasis after: orthotopic transplantation of cancer cells into the inguinal mammary fat pad (MFP) of 4 mice, where intravasation relies on active shedding from the primary tumour into the vasculature, followed by extravasation into the lungs; and intravenous tail vein injection (IV) of cancer cells into 5 mice, where the intravasation process is bypassed. Here, 100,000 combinatorial SVT cells from the same cell line were injected into multiple mice to enable the differences in colour distributions and frequencies to be compared across animals and assays.

In both IV-injected mice (Fig. 3e–i) and MFP-injected mice (Fig. 3j–m), the number of colours and their general diversities were very similar (Fig. 3n). Furthermore, analysis of the Shannon Diversity Index, taking into consideration the number of BPs and their evenness, didn’t show any statistical difference between groups (Supplementary Fig. 3).

**Fig. 4:**
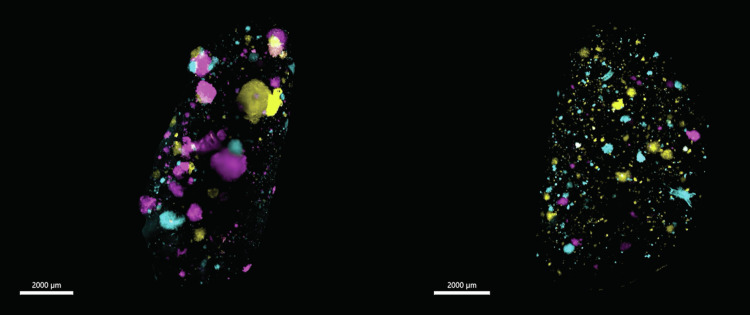
Comparison video of metastasis volumes in IV-injected and MFP-injected mice. Lung lobes acquired via light sheet microscope from IV-injected mouse (left) and MFP-injected mouse (right). SVT BPs (Cyan, Yellow Green, Magenta, Purple, Orange, White) and blood vessels (grey) are shown. Scale bar 2000 μm.

To study the number of metastases and their burden in the lungs, we separated metastatic tumours into 4 different groups according to their sizes (µm^3^), including small (1 × 10^5^ to 1 × 10^6^ µm^3^) (Fig. 3o), medium (1 × 10^6^ to 1 × 10^7^ µm^3^) (Fig. 3p), large (1 × 10^7^ to 5 × 10^7^ µm^3^) (Fig. 3q) and very large tumours (>5 × 10^7^ µm^3^) (Fig. 3r). Both IV and MFP-injected mouse assays had similar numbers of metastases (Fig. 3s, Supplementary Table 1). However, IV-injected mice had a greater number of very large metastases (median = 4), with MFP-injected mice having very few or no very large metastases (median = 0) (Fig. 3r, Supplementary Fig. 4a, Fig. 4). This data suggests that some IV-metastases are likely to be larger because they are injected directly into the blood stream, where they bypass intravasation and are seeded at much earlier timepoints than those derived from MFP-injection. However, we did not observe any significant difference regarding the time to ethical endpoint for IV- and MFP-injected mice (Supplementary Fig. 4b). This was due to a comparable overall metastatic biomass between IV and MFP-injected mouse assays (Fig. 3t). These observations were similar to a previous study using genetic barcoding^30^, although more barcodes were detected in the lungs of IV injected mice compared to the MFP group.

We then took advantage of the optical barcoding technology to study intra-metastatic heterogeneity in both groups. We showed that regardless of the mode of injection, there were significantly fewer polychromatic metastases than monochromatic metastases in the mouse lung tissue (Fig. 3u). Despite being less abundant, the polychromatic metastases showed a trend of being larger in volume compared to the monochromatic metastases in the lungs of both IV and MFP-injected mice (Fig. 3v). These findings conform with earlier studies undertaken in a transgenic MMTV–PyMT mouse model^12^ and a human cell line MDA-MD-231 and PDX MFP-injected mouse model^11^.

### A model for studying the relationship between the vascular network and metastatic dynamics in the lung

As blood vessels are a major component of the tissue microenvironment and may structurally and functionally contribute to polychromatic metastatic formation, we used vessel casting as a proof of concept to measure morphological features of the vascular network in relation to lung metastases.

**Fig. 5:**
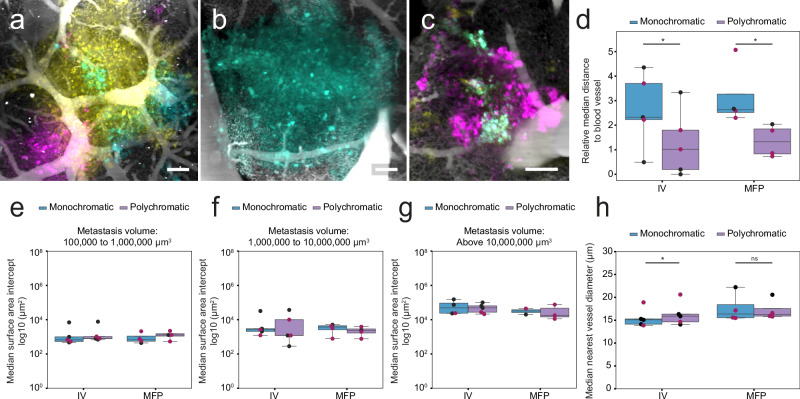
Vessel distance to metastases, median vessel surface area intercept and diameter are correlated with polychromatic metastases. **a**–**c** Representative lung SVT metastases encasing or near to blood vessels. Scale bar 100 μm. **d** Median distances of monochromatic and polychromatic metastases (relative to metastasis size) to nearest vessel in IV-injected and MFP-injected mice. Paired *t* test, *P = 0.010600 and 0.047755, respectively. **e**–**g** Median surface area intersect of monochromatic and polychromatic metastases touching blood vessels in IV-injected and MFP-injected mice. Median surface area intercept of metastases with volume **e** 100,000 μm^3^ to 1,000,000 μm^3^ (100–1000 cells, small), **f** 1,000,000 μm^3^ to 10,000,000 μm^3^ (1000–10,000 cells, medium), **g** above 10,000,000 μm^3^ (>10,000 cells, large). **h** Median nearest vessel diameter of monochromatic and polychromatic metastases in IV-injected and MFP-injected mice. Paired *t* test, *P = 0.040485, ns not significant. All box plots show the median and interquartile range (IQR), with lines representing 1.5*IQR. *n* = 5 mice for IV and *n* = 4 mice for MFP from 4 independent experiments, except for (**g**), where metastases > 10,000,000 μm^3^ were not present in every mouse. In (**g**) the number of data points is as follows: IV monochromatic (*n* = 4 mice), IV polychromatic (*n* = 5 mice), MFP monochromatic (*n* = 2 mice), and MFP polychromatic (*n* = 3 mice). The number of monochromatic metastases analysed for IV-injected mice ranged from 384 to 5300, and for MFP-injected mice ranged from 1057 to 3196. The number of polychromatic metastases analysed for IV-injected mice ranged from 21 to 419, and for MFP-injected mice ranged from 58 to 219. In (**e**–**g**), subsets of metastases were analysed based on metastasis volume. For one experiment, mice from the IV and MFP groups were injected in parallel (highlighted by the burgundy-coloured dots).

First, we examined whether the distance of a metastasis to its nearest vessel was correlated with polychromatic metastases. To investigate this, we assessed how monochromatic and polychromatic metastases were distributed around blood vessels in IV-injected and MFP-injected mice (Fig. 5a–c). Regardless of whether cells were injected IV or in the MFP, we found that polychromatic metastases were closer to blood vessels than monochromatic metastases, independently of their size (Fig. 5d, Supplementary Fig. 5a, b).

Next, of the metastases that were closely associated with a blood vessel, we measured the surface area of the metastasis that was in direct contact with the surface of a blood vessel. We observed that there was no difference in the median surface area intersection of monochromatic and polychromatic metastases across metastases of all volumes (Fig. 5e–g).

Finally, to determine if nearest vessel diameter could also provide insight into invasion mechanisms, we investigated differences in the diameter of the nearest vessel between monochromatic and polychromatic metastases in IV-injected and MFP-injected mice. Our measurements on whole lung lobe suggested that polychromatic metastases were on average, nearer to larger blood vessels than monochromatic metastases in IV-injected mice. In contrast, there was no significant difference in MFP-injected mice (Fig. 5h, Supplementary Fig. 5c).

## Discussion

The interaction between blood vessels and cancer cell populations plays a crucial role in metastasis, as the vascular network contributes to cancer cell dissemination to distant sites^31^. Here, we developed a novel experimental and computational pipeline using optical barcoding, vessel casting, tissue clearing and light sheet microscopy to detect BPs and vasculature in wholemount tissues. In the presented use case, our pipeline was applied to the analysis of lung metastases and their spatial relationship with blood vessels, for both IV-injected (experimental metastasis assay) and MFP-injected (spontaneous metastasis assay) mice. Our pipeline has been used as a proof-of-principle in a widely used breast cancer model to study late-stage metastasis, but it can also be applied to other models, such as syngeneic models or patient derived xenografts, or can be used to investigate earlier stages of metastatic spread (i.e. the extravasation of single cells or clusters of cells).

When performed on the entire lung lobe, the semi-automated analysis pipeline provides a 3D view of the metastatic landscape, where both the architecture and organisation of tissues remain intact. This is critical for studies on cellular heterogeneity as many organs are asymmetrical, where the organisation at the centre of the tissue is structurally different to peripheral regions (e.g. lymph nodes, kidneys, lungs). Furthermore, cell types and populations vary across tissue volumes (e.g. the cells lining the airways, which differ to those that comprise an alveolar unit)^32^. Hence, analyses performed on whole-mount tissues offer a better quantitative representation of cancer cell heterogeneity. An important feature of the analysis pipeline described here is that it can be applied to very large datasets and to a variety of measurements (object volumes and diameters, object-to-object distances and surface area intersects).

Using this computational approach, we found that monochromatic and polychromatic metastases differed in their overall number, size and distances to blood vessels, in both experimental and spontaneous metastasis assays. Monochromatic metastases were significantly more frequent than polychromatic metastases in lung tissue, however polychromatic metastases trended towards being larger in size. This has been previously shown in various mouse models, indicating that polychromatic metastases could have a growth advantage over monochromatic metastases, due to clonal cooperation^33,34^. We also found that polychromatic metastases were nearer to blood vessels than monochromatic metastases, independent of the mode of injection. One hypothesis for this is that proximity to blood vessels enables increased cancer cell co-recruitment for polychromatic metastasis generation. Alternatively, polychromatic metastases may be formed from heterogeneous circulating tumour cell (CTC) clusters that have extravasated from nearby vessels^35^. Previous studies have shown that CTCs that reach narrow vessels will either: 1) extravasate into the surrounding tissue, through a ‘sieve’ effect; or 2) squeeze their way through the capillary network and migrate to a distant site^36^. The observation that more polychromatic metastases are closer to larger vessels in IV injected mice, suggests that CTC clusters, which are believed to contribute to polychromatic metastases^37^, may preferentially extravasate via these vessels.

A limitation of our study is that BPs were not derived from single clones, and therefore, we cannot exclude the possibility that monochromatic metastases (of the same colour) contain genetically distinct cells. In addition, polychromatic metastases (of several colours) may contain cells that are genetically similar. Repeating these experiments with SVT barcoded single cell derived clones would deliver valuable biological insights about clonal heterogeneity and the molecular mechanisms involved in clonal cooperation and competition.

In summary, we demonstrate that LeGO-3D, combining optical barcoding, vessel casting and 3D imaging, allows an investigation of how metastatic cancer cells are distributed to secondary sites such as the lung, and their complex interplay within the tumour microenvironment of large whole-mount tissues.

## Methods

### Cell culture

MDA-MB-231 cells, isolated from the pleural effusion of an invasive ductal carcinoma patient (ATCC), and HEK-293T cells, derived from an embryonic kidney (ATCC), were cultured in Dulbecco’s Modified Eagle Medium (DMEM) supplemented with 1% (v/v) Pen/Strep and 10% (v/v) foetal bovine serum (FBS). All cell lines were maintained in a humidified incubator at 37 °C with 5% CO_2_.

### Lentivirus production, purification, and titration

The LeGO vectors were a gift from Boris Fehse^18^^,19^. Lentiviruses were generated using embryonic human kidney-293T cells and FuGENE HD transfection reagent (#E2311, Promega) mixed with three LeGO expression vectors (LeGO-S2, Addgene, #85211; LeGO-V2, Addgene, #27340; LeGO-T2, Addgene, #27342) and packaging plasmids (pCMVR8.74, Addgene, #22036; pMD2.G, Addgene, #12259). Cells were cultured in serum-free Opti-MEM (#31985070, Thermo Fisher Scientific) during all transfection and virus production steps. After 48 h, the supernatant was filtered and concentrated with Amicon Ultra-15 centrifugal filters Ultracel – 100 K (Merck, UFC910024). The virus was then titrated, and the proportion of infected cells were measured using flow cytometry. The following formula was applied: Transduction Units/mL = (Number of cells transduced × % fluorescent)/(Virus volume in mL).

### Establishment of the SVT-labelled MDA-MB-231 cells

MDA-MB-231 cells were infected with a normalised mixtures of viruses produced as described previously. The amount of virus used was empirically determined to obtain about 61% of positive cells, allowing an optimum colour distribution. One hundred thousand positive cells were sorted and plated in one well of a 6 well plate. The cells were amplified until reaching confluence in a T75 flask and resorted for positives cells to eliminate any potential negatives cells carried over from the first sort. The cells were plated back on a T75 flask and once they reached confluency, they were frozen and used for subsequent experiments.

### Mouse models

Female NOD.Cg-*Prkdc*^*scid*^ *Il2rg*^*tm1Wjl*^/SzJ (NSG) mice, aged 6-10 weeks old were used for this study. Mice were monitored twice weekly at minimum, including measurement of tumour size. During monitoring, clinical signs of metastasis were assessed, and both IV-injected and MFP-injected mice were humanely euthanised when they reached ethical endpoint (based on clinical score assessment, e.g. weight loss). All experiments were approved by the Austin Animal Ethics Committee and conducted in accordance with the National Health and Medical Research Council Guidelines.

In addition, wildtype Flk1-GFP mice were used for experiments^26^, which were approved by the WEHI Animal Ethics Committee.

### Intravenous injection and mammary fat pad transplantation of SVT-labelled cancer cells

For intravenous injections, mice were placed under infra-red heat lamp for 3 minutes, and then transferred to a restraint. After cleaning the tail with 70% ethanol, the cells, which were resuspended in a volume of ~100 μl in PBS, were injected into the lateral tail vein. For intra-mammary fat pad injection, mice were anesthetised with isoflurane then an incision was made in the skin adjacent to the 4^th^ inguinal mammary fat pad. The fat pad was held with forceps, and the cells, in a volume of 10 μl, suspended in 75% TX buffer (PBS, FCS and trypan blue) and 25% Matrigel (Corning), were injected, then the skin was sutured closed.

### Tumour resection

Tumours were measured 2–3 times weekly, and when they reached the desired volume (200–500 mm^3^), the tumours were resected. For resection surgery, mice were anesthetised with isoflurane, then a small incision was made in the skin adjacent to the tumour. The connective tissue and excess mammary fat pad was cut away to release the tumour from inside the mouse, then sterile cotton tips were used to clean the resection area, ensuring no tumour cells were left behind, before the incision was sutured closed.

### Perfusion and organ harvesting

Mice were humanely euthanised with Isoflurane (Baxter, AHN 3637) prior to transcardial perfusion with PBS and 4% paraformaldehyde (PFA) (10 mL 16% Formaldehyde Solution (w/v) (Thermo, 28906), 30 mL PBS), via the left ventricle. Two percent Porcine Skin Gelatine (Sigma, G1890-100G) in PBS was brought to boil and cooled to 40 °C. BSA-A647 (500 μg, Thermo, A34785) was added to 20 mL gelatine and filtered prior to use. Mice were perfused with filtered gelatine/BSA-fluorophore, their hearts were clamped, and then they were immediately placed in ice water for at least 15 min for gel setting (Tsai et al., 2009). Brains and lungs were dissected for post-fixation. Organs were fixed in 1% PFA overnight for vibratome sectioning or 4% PFA for 4 h for wholemount imaging.

### Single-cell suspension preparation

Tumours and tissues were collected from mice, then minced into a paste with a scalpel blade. The paste was transferred to a falcon tube containing Tumour Digestion Medium (collagenase IA (300 U/ml) (Sigma-Aldrich), hyaluronidase (100 U/ml) (Sigma-Aldrich), and deoxyribonuclease I (DNase I) (100 U/ml) (Worthington) in DMEM/F12 + glutamax (ThermoFisher Scientific) supplemented with 5% foetal bovine serum (FBS) (Moregate Biotech). The tube was incubated at 37 °C with vigorous agitation for 30–45 min. The digestion was quenched with PBS containing 2% FBS, then passed through a 70 μm sieve, spun at 500 g for 5 min, then the pellet was resuspended in PBS/2% FBS, and passed through a 40 μm sieve, before spinning at 500 g for 5 min. The subsequent pellet containing single cells was resuspended in freeze medium (10% DMSO in 90% FBS) for storage or immediately processed.

### Flow cytometry

Cells were prepared for flow cytometry by resuspending the cell pellet in FACS buffer (PBS/2% FBS) containing 1:100 DRAQ7 (Abcam). DRAQ7 was used for dead cell exclusion.

Samples were sorted using a BD FACSAria III cell sorter with BD FACSDiva 8.0.2 software (Becton Dickinson and Company, BD Biosciences, San Jose, CA, USA), equipped with four lasers and capable of detecting up to 17 parameters. Samples were analysed using the following excitation lasers and emission filters: tSapphire (405 nm; 510/50), Venus (488 nm; 530/30), DRAQ 7 (633 nm; 710/50), tdTomato (561 nm; 582/15). For sorting, a 100-μm nozzle tip was used with a sheath pressure of 138 kPa (20PSI) and a drop drive frequency of 30 kHz. BD FACSFlow (BD Biosciences, San Jose, CA, USA) sheath fluid was used. The sample and collection tubes were maintained at 4 °C.

Samples were analysed using a BD FACSymphony A3 cell analyser with BD FACSDiva 9.3.1 software (Becton Dickinson and Company, BD Biosciences, San Jose, CA, USA), equipped with five lasers and capable of detecting up to 30 parameters. Samples were analysed using the following excitation lasers and emission filters: tSapphire (405 nm; 525/50), Venus (488 nm; 515/20), tdTomato (561 nm; 586/15) and DRAQ 7 (633 nm; 710/50).

### Vibratome sectioning and Ce3D tissue clearing

Lungs, brains and tumours were fixed in 1% PFA at 4 °C overnight in the dark. The samples were washed in PBS and set into 37 °C 3% low-melt agarose (Bio-Rad, 1613111). The tissue was cut into 200 μm sections on a Leica vibratome. Ce3D (Li et al., 2017) was used to clear tissue sections (18.068 g Histodenz (Sigma, D2158-100G), 12.5 mL of 40% N-methylacetamide (40 mL N-methylacetamide (Sigma, M26305-500G), 10 mL 10x PBS (Thermo, AM9625) and 50 mL sterile water), 20 μL Triton X-100 (Sigma, 9002931), 100 μL 1-thioglycerol (Sigma, M1753-100ML)). Tissue sections were transferred to Ce3D for 2 hours at RT and then transferred to fresh Ce3D and left to clear for up to 2 days. Sections were mounted in ProLong™ Glass Antifade Mountant (Thermo, P36980, RI = 1.52). They were cured overnight before imaging on a Zeiss confocal microscope.

### Passive clarity technique (PACT) tissue clearing

PACT (Yang et al., 2014), a passive form of CLARITY tissue clearing (Chung et al., 2013) was used to clear wholemount tissue. Samples were fixed in 4% PFA in PBS for 4 h at 4 °C in the dark and then transferred to PACT monomer solution (5 mL 40% (wt/vol) acrylamide (Bio-Rad, 1610140), 5 mL 10× PBS (Thermo, AM9625), 40 mL ice-cold water, 125 mg azo-initiator (FUJIFILM, 017-19362) and kept at 4 °C in the dark overnight. The samples were placed in a vacutainer and degassed for 10 min at 4 °C. The samples were placed on a rotator at 37 °C for 4–6 h to set the acrylamide gel, before transfer into borate-buffered clearing solution (40 mL 20% (wt/vol) SDS (Sigma, 75746-1KG) in water, 20 mL 1 M boric acid buffer (61.83 g boric acid (Merck, 1001650500), 10 g sodium hydroxide (Merck, 1064980500) in 1 L of water), 40 mL water and 0.63 g sodium sulphite (Sigma, S0505-250G). The clearing buffer was changed twice daily for 2–7 days or until the samples were semi-transparent. After clearing, the samples were washed by diluting the clearing buffer (1:1) with borate-buffered wash solution (20 mL 1 M boric acid buffer, 1 mL 10% triton X-100 (Sigma, 9002931), 79 mL of water, 0.63 g sodium sulphite) thrice daily, slowly reducing the SDS concentration. Processed samples were then transferred to EasyIndex Optical Clearing Solution (LifeCanvas Technologies, EI-Z1001) (RI = 1.465) overnight, and transferred to fresh solution the next morning, before imaging on a Zeiss Lightsheet Z.7.

### Confocal image acquisition and post-processing

Tissue sections were imaged on Zeiss LSM 880 or LSM 980 confocal microscopes. A LD LCI Plan-Apochromat 25×/0.8 Imm Korr DIC objective was used to acquire tiled, z-stack images. SVT LeGO images were generated with a resolution of 0.493 μm in xy and 2 μm in z. All images were stitched in Zen Blue. Fiji was used for visualisation and presentation.

### Light sheet image acquisition and post-processing

Wholemount tissue was imaged on a Lightsheet 7 (Carl Zeiss) microscope. An EC Plan-Neofluar 5×/0.16 detection objective and 5× lightsheet objectives were used to acquire dual-side illuminated, tiled, z-stack images. SVT LeGO and vessel cast images were generated with a resolution of 1.22 μm in xy and 2.88–2.92 μm in z. T-sapphire, Venus, TdTomato and Alexa-647 excitation was captured with lasers 405 nm, 488 nm, 561 nm and 638 nm, respectively. Band pass 505–545 nm was used to capture tSapphire and Venus emitted light, band pass 575–615 nm was used to capture tdTomato emitted light and long pass 660 nm was used to capture Alexa 647 emitted light. Photobleaching and fluorophore crosstalk was kept to a minimum by optimising the imaging parameters, including fluorophore selection, laser power, light sheet illumination, and by the sequential acquisition of different channels. Fusion of dual side illuminated images and the optimisation of tissue clearing also improved signal intensity and the quality of data. In addition, imaging conditions were kept constant for each sample to minimise other forms of optical aberration, which might contribute to experimental variation. For example, a calibration was performed to optimise camera, light path and chromatic alignment (i.e. channel alignment), when setting up the instrument and prior to image acquisition. Channel alignment was also verified and corrected post-acquisition during LeGO BP correction. All images were fused and stitched in Zen Blue. Imaris, Fiji and Adobe Premier Pro were used for visualisation and presentation.

### Quantification of metastases in confocal images

Fiji (Version 1.53t) was used to quantify confocal images. SVT channels were segmented using standard thresholding. The image calculator function was used to add and subtract segmented SVT image channels, which were then analysed using the plugin ‘MorpholibJ’ to calculate the total 3D volume of each of the seven LeGO BPs. Lung cancer cell populations were corrected manually upon visual inspection. Tumour images did not undergo correction.

### Quantification of metastases and vessels in light sheet images

Fiji (Version 1.53t) was used to process light sheet series 2 (downsized) images. A median filter (radius: 2 pixels) and background subtraction (rolling ball radius: 750 pixels) was performed on each of the tSapphire, Venus and tdTomato channels. SVT and blood vessel channels were segmented in Ilastik (Version 1.4.0), a pixel classifier (Berg et al., 2019). Segmentation training was performed on three to nine planes for blood vessels and each of the three SVT channels, with annotations used to inform differences between vessels/metastases and tissue background. This training was extrapolated to the remaining image planes and a segmentation prediction was generated for each channel. Probability maps were generated, followed by binary segmented images using manual Fiji intensity thresholding. Segmented images were used to perform calculations to measure metastasis volume, nearest vessel diameter, distance of metastasis to nearest vessel, metastasis and vessel surface area overlaps, and combinatorial SVT BP distributions. A Fiji macro script was written enabling: (1) the addition of SVT channels to create an ‘all metastasis’ image; (2) calculation of the location and volume of all metastases; (3) a local thickness map (Dougherty et al., 2007) to calculate vessel diameter. Metastases were filtered for size to exclude objects that were smaller than a volume of 9000 μm^3^, the approximate volume of a single cell, to ensure noise was excluded from analysis; 4) Due to the number of metastases and size of the image data, an HPC enabled pipeline was developed to split each metastasis into an individual TIF image for manual validation and correction by a semi-automated Fiji macro with manual annotation by the expert. Manual annotation of metastatic tumours in entire lung lobes required anywhere from half a day to three days.

To examine the distribution of metastases around blood vessels, the edge-to-edge distance for metastases and blood vessel were measured. As larger metastases are more likely to have shorter distances to vessels, the measurements were normalised to the radius of each metastasis. This provides a measure independent of the size of each metastasis. As metastases are not spherical, the radius was computed by approximating a sphere of equivalent volume^38^.

The data analysis was performed in Python, using pandas (2.2.1) and numpy (1.23.4). The code and a subset of the trained Ilastik models (with corresponding training images) are available at https://github.com/BioimageAnalysisCoreWEHI/lego_analysis/tree/main/data_analysis.

### Statistical analysis

Statistical analysis was performed using independent or paired *t*-tests via the scipy package (v1.12.0). Graphs are represented as box plots or violin plots (seaborn 0.13.2), and *P* values are specified in figure legends.

## Supplementary information

Supplementary Materials

## Data Availability

Reference datasets are available from the Bioimage Archive: Bioimage Accession number - S-BIAD2145 and at 10.6019/S-BIAD2145. The full dataset supporting the findings of this study are also available from the corresponding author, K.L.R upon request.

## Code availability

The underlying code for this study is available in lego_analysis and can be accessed via this link https://github.com/BioimageAnalysisCoreWEHI/lego_analysis/tree/main/data_analysis.
