# Supplementary material for: LeGO-3D: 3D imaging of lung metastases and vascularisation using light sheet fluorescence microscopy
Source: Npj Imaging. 2025 Nov 7;3:58. doi: 10.1038/s44303-025-00111-0 (PMC12595114; doi:10.1038/s44303-025-00111-0)
Supplement: Supplementary file 2 — Supplementary Materials [file 44303_2025_111_MOESM2_ESM.pdf]

Supplementary Information

LeGO-3D: 3D imaging of lung metastases and vascularisation using light sheet fluorescence microscopy

Sabrina M. Lewis[1,2*], Jean Berthelet[3,4], Lachlan W. Whitehead[1,2], Pradeep Rajasekhar[1,2], Farrah El-Saafin[3,4], Caroline Bell[3,4,5], Shalin Naik[1,2], Delphine Merino[3,4,6], Verena C. Wimmer[1,2,6,7], Kelly L. Rogers[1,2,6,7]

1. Walter and Eliza Hall Institute of Medical Research, 1G Royal Parade, Parkville, VIC 3052, Australia.
2. Department of Medical Biology, University of Melbourne, Parkville, VIC 3010, Australia.
3. Olivia Newton-John Cancer Research Institute, Heidelberg, VIC 3084, Australia.
4. School of Cancer Medicine, La Trobe University, Bundoora, VIC 3086, Australia.
5. Austin Health, Heidelberg, VIC 3084, Australia.
6. These authors contributed equally
7. To whom correspondence should be addressed, wimmer.v@wehi.edu.edu or rogers@wehi.edu.au

* Current address: Heidelberg University Hospital, 69120 Heidelberg, Germany

Supplementary Table 1. Metastasis numbers

**Mouse**	**Assay**	**Number of monochromatic metastases**	**Number of polychromatic metastases**	**Total number of metastases**
1	IV	2682	99	2781
2	IV	384	21	405
3	IV	5300	56	5356
4	IV	2477	419	2896
5	IV	2188	168	2356
6	MFP	1322	58	1380
7	MFP	1207	219	1426
8	MFP	3196	210	3406
9	MFP	1057	89	1146

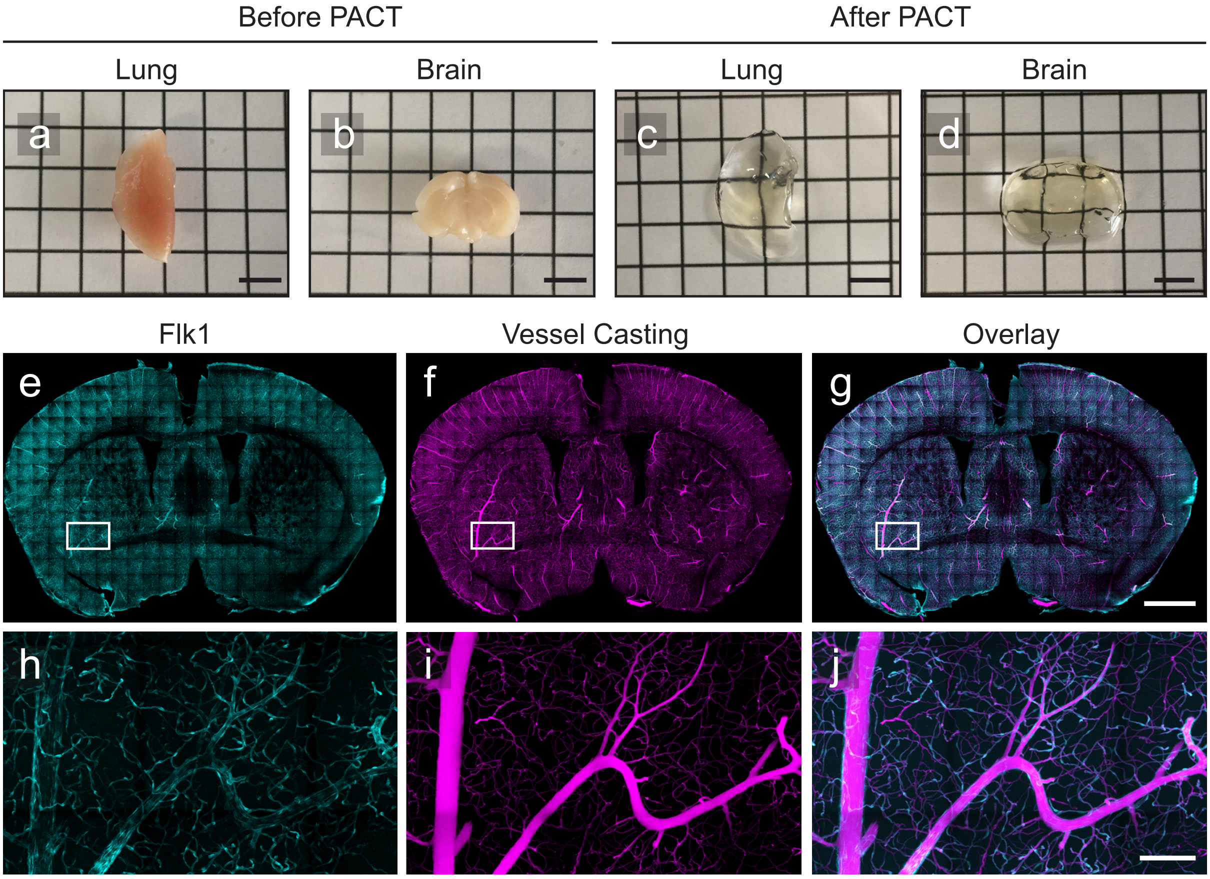

Supplementary Figure 1. PACT tissue clearing in combination with vessel casting

***(a)** Wholemount lung lobe and **(b)** 1 mm brain section before PACT clearing. **(c)** Wholemount lung lobe and **(d)** 1 mm brain section after PACT clearing. Scale bar 5 mm. **(e)** Flk1-GFP (cyan) and **(f)** vessel cast (magenta) vessels in brain image acquired via confocal microscopy. **(g)** overlaid Flk1-GFP (cyan) and vessel cast (magenta) image. Scale bar 1000 µm. **(h,i,j)** Magnified regions from white boxes in e,f,g. Scale bar 100 µm.*

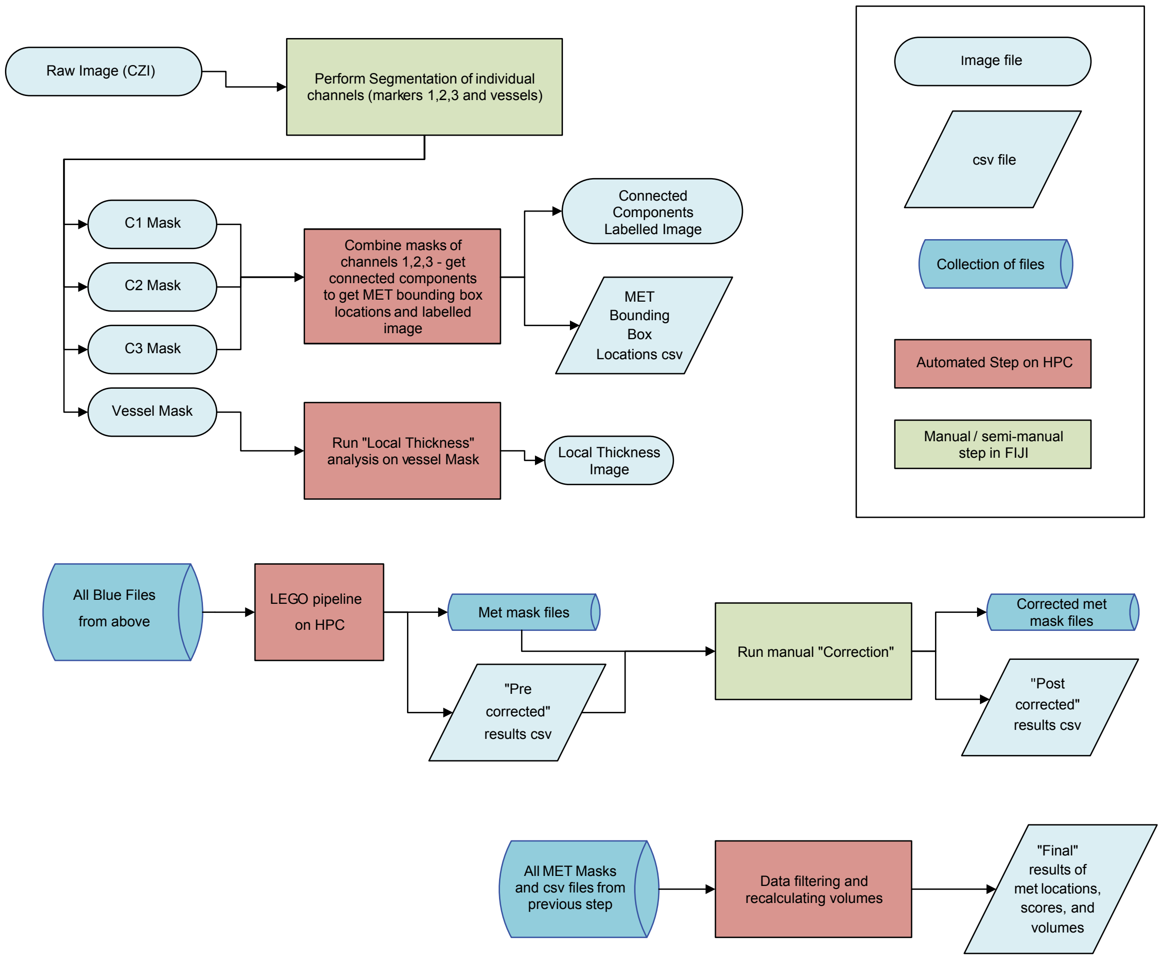

Supplementary Figure 2. Analysis flowchart using semi-automatic and automatic Fiji Macro and Python scripts.

An analysis workflow was designed that inputs raw image data (czi files) followed by segmented mask images (tif files) and outputs individual metastasis images (MET tif files), metastasis locations (MET csv files), and local thickness images from the vessel mask images (tif images). After the semi-automatic 'correction' is performed, corrected metastasis images (MET tif files) and new results files (csv files) are generated.

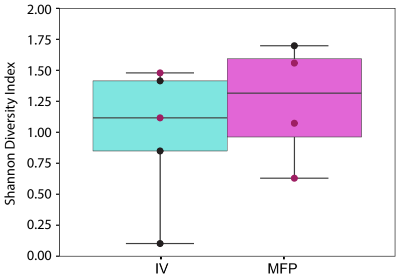

Supplementary Figure 3. Comparison of barcoded population diversity in IV and MFP-injected mice

*Shannon diversity plot in IV and MFP mice. Box plots show the median and interquartile range (IQR), with lines representing 1.5*IQR. n = 5 mice for IV and n = 4 mice for MFP from 4 independent experiments. For one experiment, mice from the IV and MFP groups were injected in parallel (highlighted by the burgundy-coloured dots).*

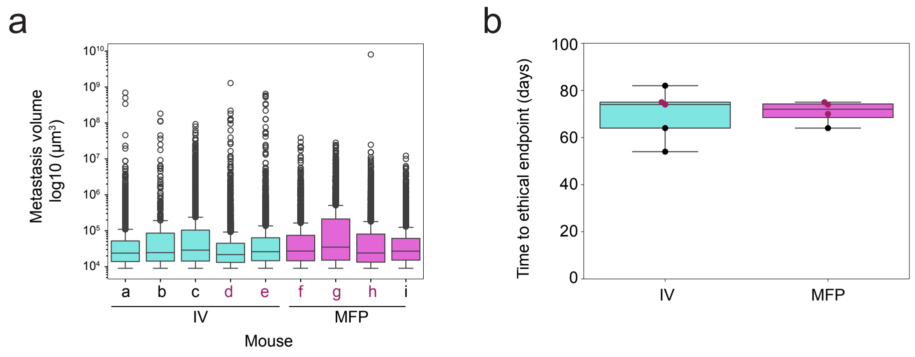

Supplementary Figure 4. Metastasis volumes and time to ethical endpoint plot for IV-injected and MFP-injected mice

(a) *Raw volumes of all metastases in independent mice. Metastases smaller than 9000 µm^3 were filtered out of final data.* ***(b)*** *Time to ethical endpoint (days) for IV-injected and MFP-injected mice from Fig. 3. In (b), n = 5 mice for IV and n = 4 mice for MFP from 4 independent experiments. All box plots show the median and interquartile range (IQR), with lines representing 1.5*IQR. For one experiment, mice from the IV and MFP groups were injected in parallel (highlighted by the burgundy-coloured dots).*

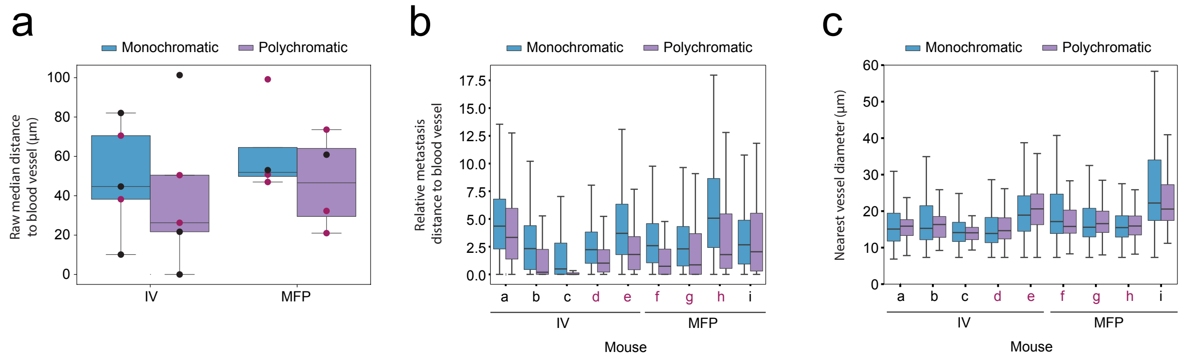

Supplementary Figure 5. Distance of monochromatic and polychromatic metastases to blood vessels

(a) *Raw median distances of monochromatic and polychromatic metastases to nearest vessel in IV-injected and MFP-injected mice. n = 5 mice for IV and n = 4 mice for MFP from 4 independent experiments.* ***(b)*** *Distances of all monochromatic and polychromatic metastases (relative to metastasis size) to nearest vessel in individual mice.* ***(c)*** *Nearest vessel diameters of all monochromatic and polychromatic metastases (relative to metastasis size) to nearest vessel in individual mice. All box plots show the median and interquartile range (IQR), with lines representing 1.5*IQR. For one experiment, mice from the IV and MFP groups were injected in parallel (highlighted by the burgundy-coloured dots).*
